# Outcome of Islanded Gastrocnemius Musculocutaneous Flap in Orthopaedic Practice

**DOI:** 10.5704/MOJ.1903.004

**Published:** 2019-03

**Authors:** MN Yusof, AA Ahmad-Alwi

**Affiliations:** Department of Orthopaedics, Traumatology and Rehabilitation, International Islamic University of Malaysia, Kuantan, Malaysia; * Department of Surgery, International Islamic University of Malaysia, Kuantan, Malaysia

**Keywords:** surgical flaps, leg injuries, wounds

## Abstract

**Introduction:** Large wounds in the leg require combination of local flaps or free flap for wound coverage. Gastrocnemius musculocutaneous flap (GMCF) allows a large wound to be covered by a single local flap. However, the conventional GMCF is often associated with donor site morbidity where the exposed soleus raphe causes poor uptake of the skin graft. Islanding the skin on the muscles allows the donor site to be closed primarily, thus avoiding the donor site morbidity.

**Materials and Methods:** Medical records of twelve patients who underwent islanded GMCF surgery from 2004 till 2018 were reviewed retrospectively.

**Results:** The mean age was 31 years old. Eight cases were with open fracture of the tibia, two degloving injury exposing the patella, one open fracture of patella and necrotising soft tissue infection. The wound size ranged from 12cm^2^ to 120cm^2^. All flaps survived. Three patients required skin grafting at the donor site while in the rest the donor sites were able to be closed primarily. Four patients developed deep infection, one healed after vacuum dressing, one after bone transport and one after split thickness skin graft. One patient ended up with below knee amputation after developing chronic osteomyelitis of the tibia.

**Conclusion:** Islanded gastrocnemius musculocutaneous flap is an effective simple alternative for coverage of large soft tissue defects from the knee to half of the leg distally with minimal donor site morbidity. Aggressive debridement of unhealthy tissue is necessary to prevent infection following wound coverage with this flap.

## Introduction

The coverage of large soft tissue defects on the leg is challenging and usually requires a combined locoregional flaps or a microvascular free tissue transfer^1^. The latter commonly performed by plastic surgeons is labour- and resource-intensive, necessitating microsurgical equipment as well as intensive post-operative flap monitoring. Gastrocnemius muscle flap is a relatively common procedure in orthopaedic practice for coverage of proximal leg wounds. The muscle, located in the superficial posterior compartment of the leg, is usually spared during injury. Although the width and reach of the flap can be increased by scoring its fascia or by detaching its origin, this spindle-shaped muscle alone cannot cover large wound at the proximal third of the leg^2-4^. However, the area of coverage can be extended from the patella down to the distal third of the leg when the skin overlying the gastrocnemius muscle is included.

One of the main problems with gastrocnemius musculocutaneous flap (GMCF) is the potential donor site morbidity, particularly when a wide skin paddle is taken, due to the exposed soleus muscle raphe and Achilles tendon that do not take skin graft very well^3^. To overcome this, we have limited the width of the skin island whenever possible and extended its length distal to the muscle to allow primary closure of the donor site.

The aim of this paper is to describe the surgical technique and to look at the outcomes related to the use of GMCF in orthopaedic surgery.

## Materials and Methods

This is a case series of twelve patients who underwent islanded GMCF surgery in our hospital from 2004 until 2018 based on our registry. Their medical records were retrospectively reviewed and the data were collected for analysis. The surgical technique was based on the concept of the musculocutaneous perforators at the leg.

**Fig. 1:**
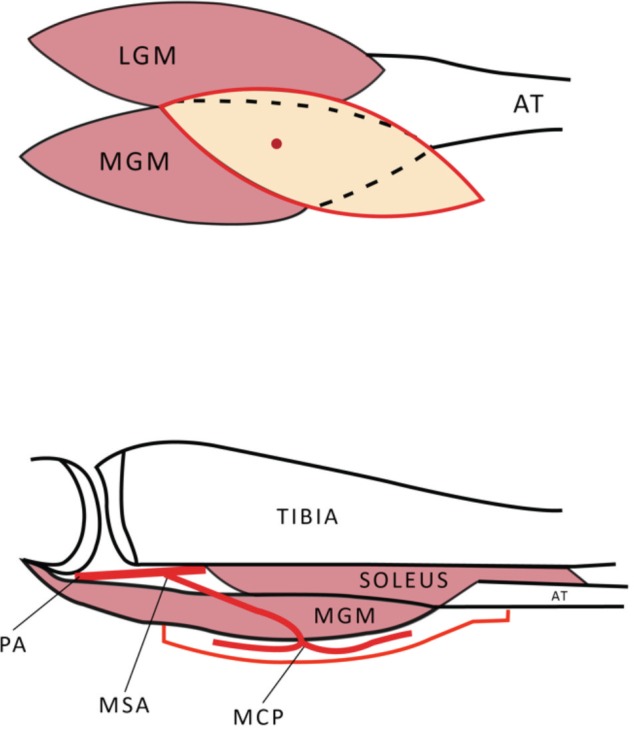
Schematic diagram showing the anatomy of the gastrocnemius musculocutaneous flap.

Gastrocnemius muscle consists of two heads, medial and lateral. Each obtains its blood supply from a dominant vascular pedicle near its origin, namely the medial and lateral sural arteries, respectively. They originate from the popliteal artery at the level of the knee joint. It is a Type I muscle flap based on the Mathes and Nahai classification which means it has only one dominant vascular pedicle^4^. Large musculocutaneous perforators from the medial gastrocnemius muscle can be found consistently at the distal half of the muscle approximately 15cm from the popliteal crease^5,6^. The perforators must be included in the skin island for the cutaneous part of the flap to survive (Fig. 1).

**Fig. 2:**
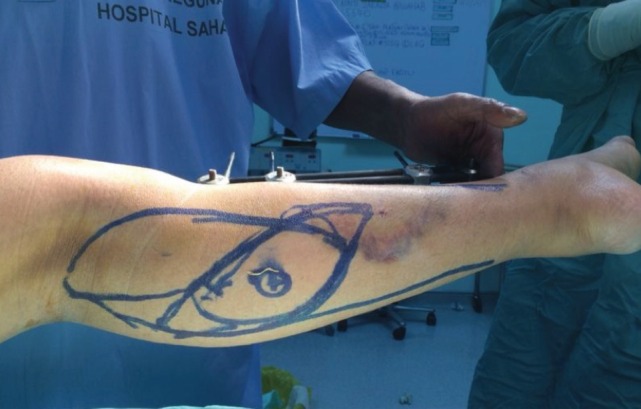
The design of the musculocutaneous flap making sure the skin island included the perforators (label X) which are located at the distal half of the medial gastrocnemius muscle.

Surgical technique for the GMCF has been well described by McCraw *et al.* The patient is positioned supine with the leg externally rotated and knee flexed^3^. A sandbag is placed beneath the contralateral hip to improve exposure if necessary. The planned skin paddle is marked. Presence of skin perforators is confirmed using a handheld Doppler ultrasound. These are usually found at the distal half of the gastrocnemius muscle in between the two heads. The flap is designed such that most of the skin paddle overlies the muscle than distal to it and the mapped perforators are included (Fig. 2).

We routinely use tourniquet with the leg partially exsanguinated by elevation. The distal segment of the skin with its deep fascia is elevated first. The skin incision is extended proximally in the midline curving medially as it approaches the popliteal fossa. The sural nerve and the short saphenous vein are retracted laterally and protected during dissection. It is our common practice to temporarily secure the skin paddle to the muscle with sutures to prevent accidental shearing during flap elevation.

**Fig. 3:**
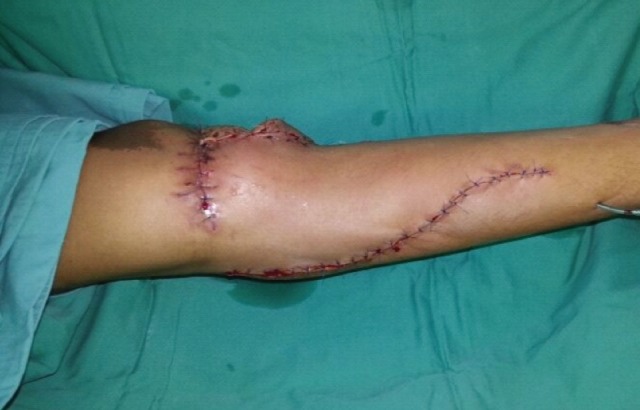
The donor site which has been closed primarily.

Approximately 1-2cm of the Achilles tendon distal to the medial gastrocnemius muscle tendon is included to provide strong tissue for anchorage at the recipient site. The medial gastrocnemius muscle can then be easily separated from the deeper soleus muscle by blunt or finger dissection in a distal to proximal direction. The two bellies of the gastrocnemius muscle are separated along the sural nerve which is the landmark that divides the two. The donor site can usually be closed primarily (Fig. 3). However, skin grafting is necessary when a large skin paddle is taken.

## Results

There were nine males and three females with a mean age of 31 years old (range: 13 to 60). The skin defects were in open fracture grade IIIB of the tibia (8 cases), degloving injury exposing the patella ligament and the patella (2 cases), open fracture of the patella (one case) and necrotising soft tissue infection (one case). Comorbidities included one case each of diabetes mellitus, hypertension, Alzheimer’s disease and HIV positive. One patient had associated sciatic nerve palsy and another had peroneal nerve palsy. The wound sizes ranged from 12cm^2^ to 120cm^2^ (Table I). Three patients had a monolateral external fixation, four had Ilizarov circular fixation, and one had plating procedures to stabilise the fractures.

**Table I:** List of patients underwent islanded gastrocnemius flap

	Age	Sex	Site	Wound size (cm)	Indication	Medical problem	Bone procedure
1	18	M	Upper 3rd of tibia	15 X 7	Open fracture grade IIIB	No	External fixation
2	32	M	Upper 3rd of tibia	6 X 5	Open fracture grade IIIB	HIV +ve	External fixation
3	15	M	Midshaft of tibia	10 X 6	Open fracture grade IIIB	None	plating
4	13	M	Knee	8 X 6	Degloving injury	None	None
5	58	F	Upper to shaft of tibia	12 X 6	Necrotising fasciitis	Diabetes mellitus	None
6	33	F	Upper 3rd tibia	10 X 6	Open fracture grade IIIB	None	Ilizarov external fixation
7	20	M	Knee	10 X 10	Infected open fracture patella	None	None
8	25	M	Midshaft of tibia	12 X 10	Open fracture grade IIIB	None	External fixation
9	13	M	Upper 3rd of tibia	12 X 8	Degloving injury	None	None
10	41	M	Upper 3rd of tibia	5 X 5	Open fracture grade IIIB	None	Ilizarov bone transport
11	44	F	Upper 3rd of tibia	10 X 8	Open fracture grade IIIB	Hypertension	Ilizarov external fixation
12	60	M	Upper 3rd of tibia	5 X 5	Open fracture grade IIIB	Alzheimer disease	Ilizarov external fixation

**Fig. 4:**
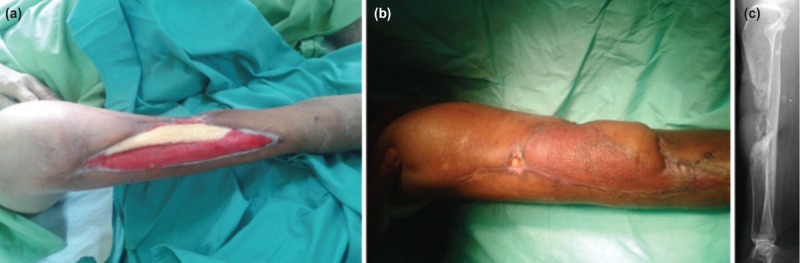
(a) A 58-year old patient with diabetes with exposed and dried shaft of tibia following debridement for necrotising soft tissue infection. (b) Patient developed persistent discharging sinus following GMCF surgery but refused further surgical intervention. (c) Plain radiograph a year after surgery showing osteolytic lesion at the midshaft of tibia secondary to chronic osteomyelitis.

All flaps survived. The donor sites were closed primarily in nine cases whilst three cases required split thickness skin grafting. Three patients developed post-operative infection which required further surgical debridement. One healed after negative pressure wound therapy, one after bone transport, and one after split thickness skin grafting. At one-year follow-up, the patient who had necrotising soft tissue infection developed osteomyelitis of the tibia and had to undergo a below knee amputation (Fig. 4).

The patient with open patellar fracture had a 10-degree extension lag because of patellectomy. One patient who was treated with Ilizarov circular fixation had a 10-degree flexion contracture of the knee and another had a 10-degree flexion contracture of the ankle. The patient with the knee contracture underwent manipulation under anaesthesia and release of the origin of the medial gastrocnemius muscle. She did not have much improvement in the range of motion but was able to continue working as a parking attendant with the local authority. The patient with ankle contracture underwent percutaneous Achilles tendon lengthening during removal of the Ilizarov circular external fixator. She had full range of motion of her ankle and was able to ambulate without support.

## Discussion

Our case series is quite small because most of the soft tissue defects were small and able to be covered using gastrocnemius muscle flap. Arnold and Mixter improvised the technique in order to gain more versatility of the gastrocnemius flap by detaching the muscle origin and scoring the overlying fascia^7^. However, this technique could only increase the size and reach of the flap by a few centimetres. Lamaris *et al* in their cadaveric study found that the reach could be increased further when a posterior midline incision was used compared to medial incision^8^. The average increase in reach was 2.02cm and total surface area of 20.3cm^2^.

McCraw *et al* introduced the concept of musculocutaneous flap which allows larger area of soft tissue coverage. This flap has been used to cover large defects around the knee and up to 5cm from the medial maleolus^3^. Cheng *et al* were able to cover defects 2cm proximal to the medial malleolus by raising the skin paddle as an “island” and cutting the muscle origin^9^. The GMCF is more reliable than a fasciocutaneous flap for coverage of a large defect because it has dual blood supply to the skin, namely from the fasciocutaneous plexus and from the musculocutaneous perforators^9^.

Complications at the donor site from GMCF are mainly related to skin graft failure when it is required. Dissecting the cutaneous part of the flap from the Achilles tendon is quite delicate. Skin graft would not take if the tendon is completely denuded of its paratenon. However, most of the time, small wounds would heal secondarily because the soleus muscle beneath the tendon has an excellent blood supply. Other complication at the donor site is skin edge necrosis when the wound is closed primarily under tension. Chung *et al* have described a gastrocnemius adipofascial flap to overcome this problem by sparing the skin^10^. Calderon *et al* have used a V-Y island gastrocnemius musculocutaneous flap to allow primary closure of the donor site^11^.

In our series, we have found that a spindle-shaped skin paddle allowed for primary closure of the donor site in the majority of cases. The empty space left after the muscle is transposed further facilitates closure of the donor site. The width of the skin paddle is dependent on the defect requirement. It is therefore important to assess the laxity of the skin to ensure that primary closure is achievable or that the paratenon on the Achilles tendon is preserved in case skin grafting is necessary.

A quarter of our patients who had infection following GMCF were related to inadequacy of debridement. This is higher than reported by Daigeler who had a 4% infection rate^12^. The success rate of treating osteomyelitis with muscle flap has been reported from 56% to 91% with poorer outcome in patients with medical morbidities and diffuse osteomyelitis^13-15^. It is sometimes difficult to judge how much tissue to removed especially during debridement of acute infection.

Some surgeons immobilise the knee in extension for two to three weeks after gastrocnemius flap because they reported higher wound complication rate with early mobilisation^16^. Reduced joint range of motion and muscle strength have also been reported by Daigeler *et al* in their series of patients who underwent gastrocnemius muscle flap^12^. We did not immobilise the knee following GMCF in this series and had not found any flap-related complications. The joint contractures and limitation in the range of motions in our series occurred in patients with severe intra-articular open fractures treated with Ilizarov circular external fixation.

## Conclusion

Gastrocnemius musculocutaneous flap is a simple and reliable solution for coverage of large wounds around the knee and proximal half of the leg. A spindle-shaped skin island design has minimal donor site morbidity when the donor site is closed primarily or when it is skin-grafted.
